# Yang/Qi Invigoration: An Herbal Therapy for Chronic Fatigue Syndrome with Yang Deficiency?

**DOI:** 10.1155/2015/945901

**Published:** 2015-02-11

**Authors:** Pou Kuan Leong, Hoi Shan Wong, Jihang Chen, Kam Ming Ko

**Affiliations:** Division of Life Science, The Hong Kong University of Science & Technology, Clear Water Bay, Hong Kong

## Abstract

According to traditional Chinese medicine (TCM) theory, Yang and Qi are driving forces of biological activities in the human body. Based on the crucial role of the mitochondrion in energy metabolism, we propose an extended view of Yang and Qi in the context of mitochondrion-driven cellular and body function. It is of interest that the clinical manifestations of Yang/Qi deficiencies in TCM resemble those of chronic fatigue syndrome in Western medicine, which is pathologically associated with mitochondrial dysfunction. By virtue of their ability to enhance mitochondrial function and its regulation, Yang- and Qi-invigorating tonic herbs, such as Cistanches Herba and Schisandrae Fructus, may therefore prove to be beneficial in the treatment of chronic fatigue syndrome with Yang deficiency.

## 1. Concepts of Yang and Qi in Traditional Chinese Medicine

Traditional Chinese medicine (TCM) views the human body as an organic entity, consisting of an assembly of various organs that function in a mutually interdependent manner [1]. “Yin/Yang Theory” is a conceptual framework of TCM. According to Yin/Yang theory, the universe is a result of a unity of opposing forces, namely, Yin and Yang. The dynamic equilibrium between Yin and Yang determines the status/phase of a given object [2]. With this philosophical concept, TCM classifies body structures, explains clinical symptoms, and guides treatment of diseases on the basis of the Yin/Yang Theory [3]. Vital substances (namely, essence, Qi, blood, and body fluid) are fundamental to life and provide the material and functional basis of the human body [1]. According to Yin/Yang Theory, functional activities of the body (such as Qi) are classified as Yang, while the material basis (such as essence, blood, and body fluids) of vital functions belongs to Yin [4].

**Figure 1:**
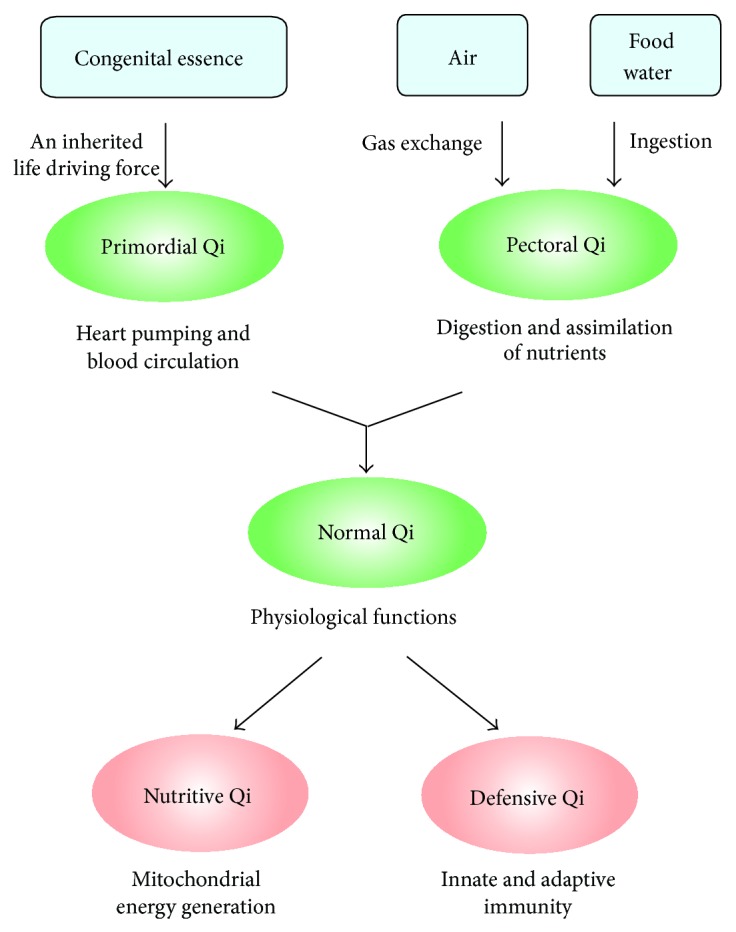
Biochemical and physiological basis of Qi function.

TCM theory states that the interaction between Yin and Yang generates Qi. Qi refers to the refined and nutritive substances flowing in the body as well as the functional status of organs and tissues. Within this framework, the complete deprivation of Qi signifies death in TCM [5]. To provide vital energy for supporting life activities, Qi flows through the meridians and nourishes the organs. With regard to the role of Qi in modulating physiological functions, Qi can be subcategorized into three functionally related types, namely, primordial Qi, pectoral Qi, and normal Qi, with the latter being subdivided into nutritive Qi and defensive Qi (Figure 1) [5]. In essence, primordial Qi which is also known as “congenital essence of the kidney” is inherited from parents and is responsible for stimulating growth and development, as well as invigorating the vital activities of organs in the body; that is, it is Yang in nature. Pectoral Qi is comprised of the “natural air” inhaled by the lungs and the “grain Qi” transformed from food and water by the spleen and stomach; that is, it is Yin in nature. The principal actions of pectoral Qi are to facilitate gas exchange in the lungs and regulate blood circulation in the heart as well as its rate of beating. Primordial Qi combines with pectoral Qi to form normal Qi (also called Zheng Qi in Chinese), which circulates in the body for supporting various body functions. The interrelationship between primordial Qi and pectoral Qi is consistent with the notion that Qi (or normal Qi) arises from an interaction between Yin and Yang. Normal Qi (generally referred to as Qi hereafter) manifests as two functions, namely, nutritive Qi and defensive Qi. While nutritive Qi nourishes the internal organs to sustain the physiological functions of the body, defensive Qi protects the body against disease-causing internal (inflammation and cancer) and external (bacteria and viruses) factors.

## 2. Biochemical and Physiological Basis of Qi Function

Over the past decades, the mitochondrion has been considered to be a central coordinator of life and death in cells by virtue of its regulatory role in both bioenergetics and programmed cell death [6]. According to TCM theory, the depletion of Qi is casually linked to death. In this regard, the concept of Qi in TCM is consistent with the vital role of mitochondria in determining life and death within the conceptual framework of Western medicine.

The mitochondrion is the “power house” of the cell, where the aerobic metabolism of fuel molecules takes place. In aerobic metabolism, acetyl-CoA, which is formed from glucose via glycolysis and oxidative decarboxylation of pyruvate, enters the Krebs cycle occurring in the mitochondrial matrix. Acetyl CoA is ultimately oxidized to carbon dioxide, with the concomitant production of the high energy reducing equivalents, NADH and FADH_2_. Both NADH and FADH_2_ then donate their high energy electrons to the mitochondrial electron transport chain that generates a proton gradient across the mitochondrial inner membrane. By utilizing the electrochemical potential energy stored in this proton gradient, ATP synthase synthesizes ATP from ADP. ATP, a molecule with high phosphoryl transfer potential (i.e., potential energy), energizes a number of endergonic reactions in the cell, particularly those supporting vital activities. During the mitochondrial electron transport process, reactive oxygen species (ROS) are unavoidably produced from the leakage of electrons, particularly from complexes I and III. The excessive production of ROS from mitochondria, under conditions of high respiratory activity and/or in the presence of threats to homeostasis, results in an increase in oxidative stress. Under conditions of severe oxidative stress, mitochondrial permeability transition pores open, with the subsequent nonspecific release of proapoptotic factors (e.g., cytochrome c and apoptosis-inducing factor), leading to caspase-dependent and caspase-independent cell death [7]. With regard to the regulation of bioenergetics and cell death, the mitochondrion can be considered as the functional unit of Qi. This postulation may explain the relatively short lifespan of erythrocytes which do not have mitochondria.

Primordial Qi, which is the primary driving force of human life in the context of TCM, can be functionally related to the pumping action of the heart that maintains the circulation of the blood throughout the body (Figure 1). Recently, by adopting the concept of “resonance,” Wang et al. have proposed a novel model for explaining how a pumping heart can propel the circulation of blood throughout the body [8, 9]. In essence, the arterial system and various organs in the body are connected by branches of arterial blood vessels. The rhythmic contraction of the heart causes vibrations in the arteries and the potential energy stored in elastic walls of these blood vessels will subsequently be transmitted through the blood stream. It is hypothesized that the pumping action of the heart can provide the arterial system with a series of harmonic frequencies of oscillations that can be transmitted to various target organs. If the natural frequency of the target organ synchronizes with one of these harmonic frequencies, the resultant resonance will facilitate the entry of blood into the organ. Despite the fact that this “resonance” model has not been generally accepted in Western medicine, it paints a picture describing how primordial Qi may work by driving the circulation of blood or Qi throughout the body. Pectoral Qi results from the combination of inhaled fresh air and ingested food “essence” (i.e., essential nutrients). The digested and subsequently absorbed nutrients are first transported to the liver for assimilation. Accordingly, the function of pectoral Qi may be related to the assimilation of ingested nutrients.

Primordial Qi (Yang) interacts with pectoral Qi (Yin) to form the normal Qi, which is comprised of nutritive Qi and defensive Qi. Nutritive Qi is responsible for nourishing visceral organs. In this regard, nutritive Qi may be related to the efficiency of tissues/cells to generate energy from nutrients, that is, the efficiency of mitochondria to generate ATP using fuel molecules (Figure 1). This postulation is strengthened by the earlier notion that the mitochondrion is regarded as the cell origin of Qi (energy). In this regard, nutritive Qi may also be Yang in nature. Defensive Qi is responsible for protecting the body against disease-causing internal and external factors. In the face of pathogen invasion, the innate immune response is elicited, during which phagocytic cells (macrophages and neutrophils) migrate to the site of invasion and engulf the invading pathogen. The engulfed pathogen is then engulfed by ROS and lysozymal vesicles inside phagocytes and is thereby degraded. The “respiratory burst” involves the NADPH oxidase-catalyzed generation of ROS in phagocytic cells. According to TCM theory, bone (marrow), blood, and body fluid, which are enriched with immune cells, are classified as Yin, suggesting that defensive Qi may be Yin in nature. Recently, age-related deterioration of immunity (“immunosenescence”) was found to be associated with oxidative stress [10], and immunosenescence is closely related to the aging process [11]. In this connection, the involvement of immunosenescence in aging is consistent with the TCM theory which states that the substantial depletion of Qi is the primary cause of aging. To safeguard immunosenescence caused by oxidative stress, immune cells are equipped with an antioxidant defense system, which is composed of free radical scavengers and antioxidant enzymes. The fortification of antioxidant defense can therefore enhance immune function and thereby indirectly invigorate the defensive Qi.

## 3. “Yang/Qi Deficiency” Disease: Chronic Fatigue Syndrome

Given the causal relationship between Yang and Qi, we adopted the term “Yang/Qi” in the subsequent discussion on chronic fatigue syndrome (CFS). According to TCM theory, Yang/Qi is the driving force of biological activities in the human body. Deficiencies in Yang/Qi display a high prevalence in the “fatigue syndrome” in humans [12]. While Yang-invigoration involves the enhancement of body function and energy metabolism in various organs, Yang deficiency is characterized by decreased metabolic activities, as evidenced, for example, by a reduction in body temperature [13]. A recent metabonomic study has shown that a severe impairment in both glucose and lipid metabolism was observed in a rat model of hydrocortisone-induced kidney “Yang deficiency” [14], which reflects a deficiency of nutritive Qi in TCM. An impaired mitochondrial functional capacity, as evidenced by reduced urinary levels of creatinine (a product of phosphocreatinine breakdown) and citrate (an important metabolic intermediated in the Krebs cycle), was also found in animals with kidney “Yang deficiency” [15]. The use of ^1^H nuclear mass resonance spectrometry and partial least squares discriminant analysis in patients suffering from Yang deficiency syndrome revealed that blood lipid parameters, the ratio of low density lipoproteins to very low density lipoproteins, lactic acid, and sugars, which are fuel molecules or metabolites of energy metabolism, were found to be unbalanced and/or abnormal [16], indicative of a dysregulation of mitochondrial energy metabolism.

**Figure 2:**
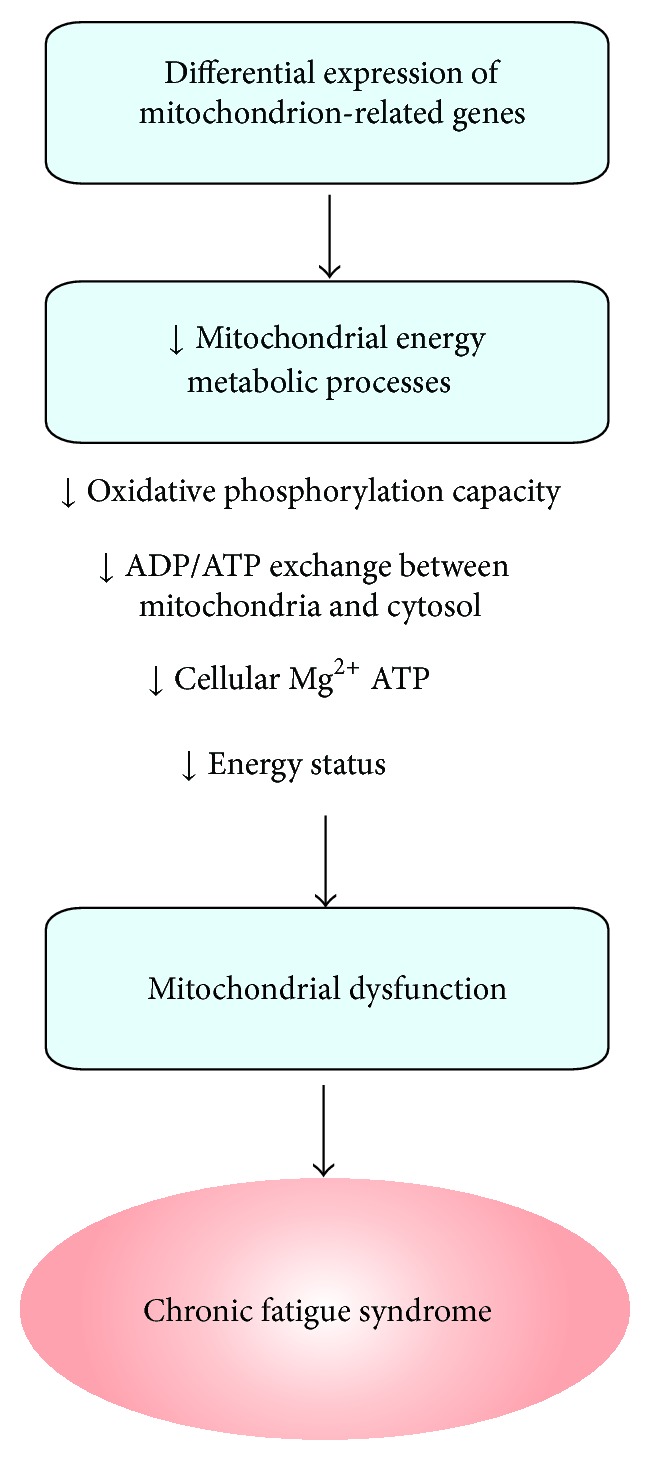
A hypothesis of mitochondrial dysfunction-mediated chronic fatigue syndrome.

Interestingly, CFS in Western medicine partially resembles the Yang/Qi deficiency-induced fatigue syndrome in TCM [17]. Unlike fatigue, which is a transient, a common self-limiting symptom, CFS is an illness characterized by a persistent (or relapsing) debilitating and clinically unexplained fatigue that leads to a substantial impairment in functional status and subsequent personal and economic morbidity [18]. Patients suffering from CFS exhibit a profound disabling fatigue for at least 6 months, which is accompanied by numerous rheumatological, infectious, and neuropsychiatric symptoms [18]. CFS is a heterogeneous syndrome, for which there appears to be a genetic predisposition, characterized by a variety of pathophysiological features including neuroendocrine abnormalities, increased susceptibility to infections, obesity, and chronic stress. Despite the diversity of these pathophysiological anomalies, mitochondrial dysfunction has been shown to be crucially involved in the development of CFS. Studies focusing on CFS-induced changes in gene expression demonstrated a differential expression pattern of mitochondria-related genes and a decrease in mitochondrial metabolic processing in CFS patients (Figure 2) [19]. It was also found that the structural integrity of mitochondria in skeletal muscle was disrupted, which was likely related to the reduction in the energy level of patients suffering from CFS [20, 21]. Cross-sectional studies using a magnetic resonance technique also identified distinctive and reproducible muscle and cardiac biogenetic abnormalities in CFS patients [22], which is a manifestation of Yang deficiency in TCM. Patients suffering from CFS displayed a marked increase in intramuscular acidosis in response to repeated exercise when compared with nonfatigued controls, suggesting an increased reliance on anaerobic metabolism as a result of the reduced mitochondrial oxidative phosphorylation capacity [23]. In addition, a clinical study investigating the association between CFS and mitochondrial function proposed that, as a heterogeneous syndrome, CFS is not necessarily associated with the impairment of mitochondrial integrity, but rather with a deficit in mitochondrial functional capacity, as evaluated by the amount of cellular magnesium-complexed ATP, the capacity of mitochondrial oxidative phosphorylation and the efficiency of ADP/ATP-exchange between mitochondria and cytosol (Figure 2). The findings demonstrated a significant correlation between the degree of mitochondrial dysfunction and the severity of CFS [21]. However, some symptoms of CFS resemble the manifestation of Yin deficiency, such as an increased sweating [24], a low grade fever [25], and a dryness of mucous membrane [26]. In addition, the dysfunction of immune system (defensive Qi, i.e., Yin in nature) was found to be associated with CFS [27, 28]. We hereby propose that CFS can be divided into 2 subcategories in terms of clinical symptoms: Yang-deficient type with mitochondrial dysfunction (cf. nutritive Qi) and Yin-deficient type with immune/body fluid regulatory dysfunction (cf. protective Qi).

A growing body of evidence has suggested the involvement of mitochondrial dysfunction in the pathogenesis of CFS with Yang/Qi deficiency. In this connection, physical exercise, which was shown to improve oxidative capacity of skeletal muscle in patients with mitochondrial myopathy [29], is being proposed as a treatment for CFS [30]. Clinical studies have revealed that graded exercise therapy and cognitive behavioral therapy produced beneficial effects in patients with CFS [31, 32]. Based on a body of clinical evidence, van Cauwenbergh et al. have summarized practice guidelines of exercise intervention for CFS patients [30]. However, Kindlon reported harmful side effects associated with the graded exercise therapy and cognitive behavioral therapy in some CFS patients [33]. Presumably, the inability of the graded exercise therapy and cognitive behavioral therapy to ameliorate the symptoms of some CFS patients may be due to the existence of Yang-deficient and Yin-deficient types of CFS. Whether or not exercise intervention is the panacea for Yang-deficient type of CFS clearly requires more extensive clinical investigation.

## 4. Yang- and Qi-Invigorating Herbs and Mitochondrial Function

In the realm of TCM, a pathological condition is caused by an imbalance of Yin/Yang status in the body. A prescription with tonic herb(s) can help to restore the balance of Yin and Yang and achieve a healthy condition. Tonic herbs are generally classified into four categories on the basis of their health-promoting actions, namely, “Yang-invigorating,” “Qi-invigorating,” “Yin-nourishing,” and “blood-enriching” herbs (Table 1). The “Qi-invigorating” and “blood-enriching” herbs possess Yang and Yin characteristics, respectively. With the notion that Yang and Qi are related to mitochondrial energy metabolism in the body, the prescription of Yang-invigorating and Qi-invigorating herbs was found to enhance mitochondrial ATP generation [34], which may be beneficial to patients with CFS of Yang-deficient type. Consistently, Yin-nourishing herbs were found to produce an immunomodulatory effects, presumably invigorating the defensive Qi (Yin) [35]. Recent studies have compared the effectiveness of various Yang-invigorating herbs in increasing mitochondrial ATP generation capacity (ATP-GC) in H9c2 cardiomyocytes* in vitro* and in rat hearts* ex vivo*. Cistanches Herba was found to increase the ATP-GC in H9c2 cardiomyocytes and in rat hearts, with the extent of stimulation being most potent among all tested Yang-invigorating herbs. Among Qi-invigorating herbs, Schisandrae Fructus has been shown to confer cellular/tissue protection against oxidative stress in rodent brain, heart, liver, and skin tissues via the enhancement of mitochondrial antioxidant status [36]. In this regard, we sought to review the pharmacological actions of two commonly prescribed Yang- and Qi-invigorating herbs in relation to their beneficial effects on mitochondrial function.

**Figure 3:**
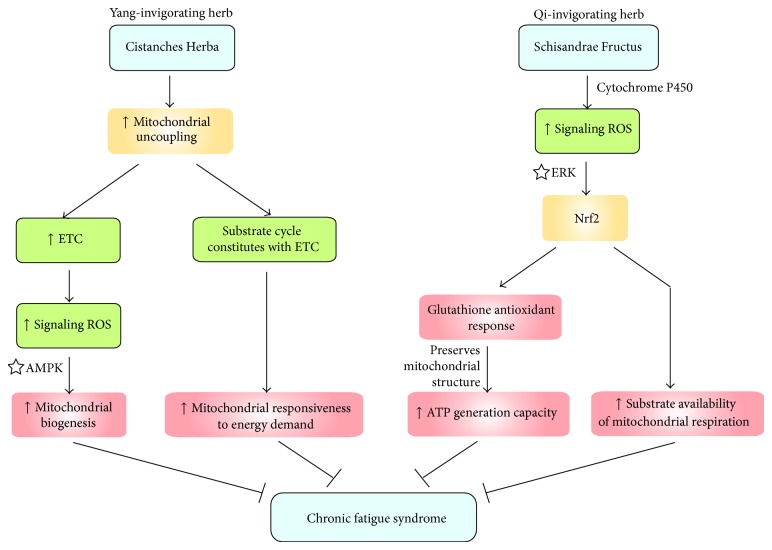
A potential treatment for chronic fatigue syndrome using Yang/Qi-invigorating herbs. ERK, extracellular signal-regulated kinases; ETC, electron transport chain; Nrf2, nuclear factor erythroid 2-related factor 2; ROS, reactive oxygen species; ☆, activation.

Cistanches Herba, one of the “Yang-invigorating” tonic herbs, was found to enhance mitochondrial respiration, as indicated by a significant increase in ATP-GC and mitochondrial state 3 respiration in H9c2 cells and in isolated rat heart mitochondria [37]. Cistanches Herba was also shown to induce mitochondrial uncoupling in both cell and animal models. The induction of mitochondrial uncoupling constitutes a substrate cycle involving the mitochondrial electron transport chain, which results in an increase in responsiveness of mitochondria to cellular energy demand [38]. The induction of mitochondrial uncoupling can in turn activate mitochondrial electron transport, which is associated with increased mitochondrial ROS production [37]. The sustained low level of mitochondrial ROS production triggers a series of cellular responses, including mitochondrial biogenesis, via the activation of AMP-activated protein kinase (AMPK) pathway [39, 40]. Taken together, the Cistanches Herba-induced increase in mitochondrial number, together with the augmented mitochondrial responsiveness to energy demand, allows sufficient energy generation to maintain physical and mental activities and thereby produce beneficial effect in CFS patients with Yang deficiency (Figure 3).

Schisandrae Fructus (namely, Wu-Wei-Zi in Chinese), the fruit of* Schisandra chinensis*, is a Qi-invigorating herb. Schisandrae Fructus possesses five tastes, namely, sweet, sour, bitter, astringent, and salty, which, according to “Five-Element Theory,” correspond to five visceral organs (spleen, liver, heart, lung, and kidney, resp.) in TCM [41]. According to TCM, Schisandrae Fructus can invigorate the Qi of these five visceral organs [41]. Over the past few decades, extensive research has focused on investigating the pharmacological activities of Schisandrae Fructus, particularly those of its polysaccharide and lignan components. Polysaccharides isolated from Fructus Schisandrae (namely, SCP-IIa and SCPP11) were found to produce an immunomodulatory effect on peritoneal macrophages and lymphocytes in mice [42, 43]. Among the lignans, schisandrin B (Sch B), the most abundant dibenzocyclooctadiene lignan in Schisandrae Fructus, was shown to possess antioxidant and anti-inflammatory activities [44]. A huge body of experimental evidence has shown that Sch B can enhance mitochondrial glutathione antioxidant status and thus protect against oxidant-induced injury under both* in vitro* [45] and* in vivo* [46] experimental conditions. Mechanistic studies have demonstrated that Sch B is metabolized by cytochrome P-450, with a concomitant production of a low level of ROS [47]. Conceivably, these “signaling ROS” then stimulate redox-sensitive ERK (extracellular signal-regulated kinases)/Nrf2 (nuclear factor erythroid-2 related factor 2)/EpRE (electrophile responsive element) signaling pathway, with a resultant expression of antioxidant proteins [47]. As proposed by the “Mitochondrial Theory of Aging,” mitochondrial dysfunction is mainly caused by cumulative oxidative damage [48]. The Sch B-elicited glutathione antioxidant response can preserve the structural integrity of mitochondria in the face of oxidative challenge, which in turn can indirectly improve the functional capacity of mitochondria, as evidenced by an elevation of ATP-GC in Sch B-treated mice [49]. These findings therefore suggest that the Qi-invigorating action of Sch B may also be beneficial in patients suffering from CFS with Yang-deficiency (Figure 3). In addition, the activation of Nrf2 by Sch B can not only enhance antioxidant defense components and reduce the extent of inflammation [44, 45], but also produce a positive impact on cellular bioenergetics by controlling substrate availability for mitochondrial respiration [50].

## 5. Conclusion

Based on the crucial role of mitochondria in energy metabolism, we propose an extended view of Yang and Qi in the context of mitochondrion-driven cellular and body function. Yang and Qi likely connote mitochondrion-driven biological processes in the human body. The manifestation of Yang/Qi deficiencies in TCM is in common with CFS of Yang deficient type, for which a huge body of clinical evidence has accumulated linking mitochondrial dysfunction to CFS. By virtue of their ability to enhance mitochondrial function and its regulation, Yang- and/or Qi-invigorating herbs, such as Cistanches Herba and Schisandrae Fructus, respectively, may prove useful for the treatment of CFS with Yang deficiency. Moreover, the astringent and immunomodulatory actions of Fructus Schisandrae may also be beneficial to CFS patients with Yin deficient symptoms such as increased sweating, dry mouth, and immune dysfunction. Future clinical studies on Cistanches Herba and Schisandrae Fructus or their combination in CFS patients, particularly those with Yang deficiency, are therefore warranted.

## Figures and Tables

**Table 1:** Different categories of Chinese tonic herbs.

Yang-invigorating	Qi-invigorating
Eucommiae Cortex	Schisandrae Frutus
Psoraleae Fructus	Ziziphi Fructus
Cistanches Herba	Astragali Radix
Cynomorii Herba	Codonopsis Radix
Epimedii Herba	Fici Radix
Dipsaci Radix	Ginseng Radix
Morindae Radix	Glycyrrhizae Radix
Cibotii Rhizoma	Pseudostellariae Radix
Drynariae Rhizoma	Quinquefolii Radix
Cuscutae Semen	Atractylodis Rhizoma
	Dioscoreae Rhizoma

Yin-nourishing	Bood-enriching

Ligustri Fructus	Lycii Fructus
Dendrobii Herba	Mori Fructus
Ecliptae Herba	Testa Dolichoris
Asparagi Radix	Loranthi Ramulus
Ophiopogonis Radix	Angelicae Radix
Oryzae Radix	Polygoni Radix
Polygonati Rhizoma	Rehmanniae Paraparata Radix
Prinsepiae Semen	Polygonati Rhizoma
	Sesami Semen
